# New and Non-Official Remedies

**Published:** 1911-10

**Authors:** 


### New and Non-Official Remedies

### I. Compounds of Metals

THE American Medical Association has published a large pamphlet of eighty pages, descriptive of the articles accepted by the Council of Pharmacy and Chemistry, up to January 1, 1911. The list contains some five hundred and forty titles and is well worth reading in its entirety but we have endeavored to condense it for more ready reference.

Omitting synonyms, simple Galenical or pseudo-Galenical preparations, mere proprietary mixtures and trade names for official drugs and chemic substances long known, such as urea, dextrose, laevulose, picric acid, and the like, animal extracts and germ products, the list is reduced by about half. We omit these last two groups for the following reasons: It is now possible to secure a dessication, extract or, in a few instances an active principle of nearly every conceivable organ, including as active principles, familiar and unfamiliar ferments. For nearly every infectious process, it is also possible to secure either a vaccine, i.e., a culture, mitigated or killed, a germ extract, or a serum. Both of these groups of remedial agents are under constant discussion and are subject to frequent improvements.

So far as practicable, the remainder of the list will be discussed under headings corresponding to established, official drugs, for which the newer preparations are claimed to be less objectionable or more potent substitutes.

### ARSENIC.

Arsanilic acid is arsenic acid with one hydroxyl group replaced by phenylamine. It is employed in the following derivatives:

Sodium acetyl arsanilate (Arsacetin.)

Sodium arsanilate. Atoxyl and Soamin, differing in water of crystalisation and corresponding respectively to 26 and 22 per cent. of arsenic. The dose of these preparations ranges from one to 20 centigrams (1-6—4 grains). All are supposed to be less toxic than official forms of arsenic.

Arseno-ferratin is the sodium salt of an albuminate of arsenic and iron.

Arsen-triferratin is iron arseno-paranucleate. The dose of these two preparations is 30 to 50 centigrams (5—7½ grains).

Ferric arsenite (soluble by addition of ammonium citrate, 1.06 per cent. of arsenic 3—6.5 centigrams (½—1 grain).

Sodium dimethyl-arsenate (Sodium Cacodylate 2½—12 centigrams (1-3—2 grains).

### BISMUTH.

Bismuth subcarbonate. Cremo-bismuth is an 8.66 per cent. suspension of finely precipitated bismuth subcarbonate. Lac bismo is a 4 per cent. suspension of mixed bismuth subcarbonate and hydroxid.

Bismuth iodo-subgallate (Airol) externally.

Bismuth methylen-digallate, (Bismal) 10 to 30 centigrams (1½ to 5 grains).

Bismuth bitannate (Tannismuth) 30—60 centigrams (5—10 10 grains).

Bismuth and lithium citrate (soluble) 10 to 30 centigrams (1½ to 5 grains).

Bismuth beta-naphtholate (Orphol) 1—5 grams (15 to 75 grains.

Bismuth bitannate (Tannismuth) 30—60 centigrams (5-10 grains).

Bismuth tribromphenolate (Xeroform) (nearly 50 per cent. of bismuth oxid) 1—3 grams per day (15—45 grains) also externally as surgical powder.

### BARIUM.

Barium chlorid. Highly toxic. Stimulant to unstriped and cardiac muscle. Dose 6—30 milligrams (1-10—½ grain).

### COPPER.

Copper citrate (dosage and strength about as for sulphate.)

Cupro-hemol see under Iron.

### MANGANESE.

See under, iron, bromine, iodine, and the like.

### OSMIUM.

Osmium tetroxid, to produce degeneration of nerves in obstinate painful affections, ½—1 c.c. of ½ per cent. solution, fresh.

### THORIUM.

Thorium nitrate, radio-active, 20 per cent. solution neutralized with ammonia and inhaled for phthisis, and the like.

### IRON (See under arsenic.)

Sodium ferri-albuminate (Ferratin) 50 centigrams (7½ grains).

Ferrous lactate 6—130 centigrams (1—20 grains.)

Iron and manganese peptonate (Ferro-mangan) 4—15 c.c. of solution (1—4 drachms).

Ferric ichthyol (Ferrichthyol) 1—2 grams (15—30 grains).

Ferripyrine or Ferropyrine, ferric chlorid 36 per cent., antipyrine 64 per cent. 30 centigrams to 1 gram (5—15 grains .

Ferric paranucleinate (Triferrin) (22.5 per cent. iron, 2.5 per cent. phosphorus). 30 centigrams (5 grains).

Haemaboloids, liquid containing 4 per mille of iron, variously combined with proteins and nucleproteins; 4 per cent. bone marrow extract. Dose 15 c.c. (½ ounce).

Haemoglobin 30 centigrams to 2 grams (5—30 grains).

Reduced haemoglobin (Hemol) 12—50 centigrams (2—7½ grains).

Hemogallol (Haemoglobin reduced by pyrogallol) 25—50 centigrams (4—7½ grains).

Haemoglobin 30 centigrams to 2 grams (5—30 grains).
centigrams (4—7½ grains).

Cupro-hemol (Hemol combined with copper, corresponding to 4 per mille iron, 2 per cent. copper, 12—50 centigrams (2—7½ grains).

Ovoferrin (Iron Vitellin) solution, 4 per mille of metallic iron. Dose 8—15 c.c. (2—4 drachms).

### SILVER.

Silver-nitrate-gelatose (Albargin) 13—15 per cent. of silver.

Ethylene-diamin silver nitrate (Argentamin) 10 per cent. of silver nitrate.

Silver mythylen-nucleinate (Sophol) 20 per cent. silver (2—5 per cent. solutions).

Silver casein (Argonin) 4.28 per cent. of silver.

Silver vitellin (Argyrol) 20—25 per cent. of silver.

Silver proteinate (Novargan 10 per cent. of silver.) (Protargol 8.5 per cent. of silver).

Silver sulph-ichthyolate (Ichthargan) 30 per cent. of silver.

Collargol (Colloid silver) 85 per cent. silver.

The above are considered less toxic and more efficient than ordinary silver salts. Excepting collargol, they are commonly used for injections, and the like, in a strength corresponding to 1-5—2 per cent. of the silver content, but with various important exceptions. Collargol may be given internally in a dose up to one grain; 2—5 per cent. intravenously; 2—15 per cent. in injections, and the like, the ordinary Crede ointment being 15 per cent.

Silver citrate, locally full strength, injections 1: 10,000—1:2,000.

Silver lactate, (irritating locally in full strength), injections 1:100—1:1,000.

MERCURY.

Mercuric benzoate (approximately equivalent in dosage and external strength to mercuric chlorid).

Mercuric cyanide (same note).

Mercuric oxycyanide (same note).

Mercuric salicylate (same note).

Mercuric succinimide 1—1½ centigrams (1-6—¼ grain by mouth), ½—1 c.c. of 2.5 per cent. solution by daily injection.

Mercury nucleinate (Mercurol) 10 per cent. metallic mercury. 3—12 centigrams (½—2 grains).

Mergal (1 part mercuric cholate, 2 parts albumin tannate) 4.4 per cent. metallic mercury. 15 centigram capsule (½ grains).

Mercuric sulphate-ethylene-diamine (Sublamine) 44 per cent. metallic mercury. (Strength for external use and internal irrigation about as for mercuric chlorid, 3—4 per cent. solution hypodermatically).

Colloid calomel (Calomelol) (Internally and externally, in ointment or diluted with starch, and the like, about as calomel. Protect from light).

Potassium mercuric iodid, a proprietary preparation in tablet of 113 milligrams (1¾ grains), practically identical with the time-honored solution of mercuric iodid in potassium iodid.

MAGNESIUM.

Magnesium stearate (Dolomol) dressing powder.

ALUMINUM.

Aluminum naphthol sulphonate (Alumnol) powder or solution, ½—20 per cent. according to location, etc.

ZINC.

Zinc permanganate, 1:4,000 locally.

Zinc peroxid 10 per cent. ointment up to full strength as dusting powder.

Zinc sulphanilate (Nizin) 1-5—1 per cent. as astringent and antiseptic, according to location, eye, urethra, etc.

